# Correlation between Altmetric Attention Scores and citation scores across the high impact-factor journals each in Medicine, Surgery, and Anaesthesia

**DOI:** 10.1016/j.bja.2024.09.034

**Published:** 2024-12-10

**Authors:** Amanda Koh, Christopher A. Lewis-Lloyd, Tiffany Wong, Dileep N. Lobo

**Affiliations:** 1 Nottingham Digestive Diseases Centre, Division of Translational Medical Sciences, School of Medicine, University of Nottingham, Nottingham, UK; 2 National Institute for Health Research (NIHR) Nottingham Biomedical Research Centre, Nottingham University Hospitals NHS Trust and University of Nottingham, Queen's Medical Centre, Nottingham, UK; 3 MRC Versus Arthritis Centre for Musculoskeletal Ageing Research, School of Life Sciences, University of Nottingham, Queen's Medical Centre, Nottingham, UK; 4 Division of Surgery, Perelman School of Medicine, University of Pennsylvania, Philadelphia, PA, USA

**Keywords:** altmetrics, Altmetric Attention Score, bibliometrics, citation count, journal impact factor

## Abstract

**Background:**

Citation scores (CS) are traditionally used to measure the impact of scientific publications. Altmetric Attention Scores (AAS), in contrast, consider the digital dissemination of articles across social media platforms to track their audience reach. In this cross-sectional study, we aimed to determine the correlation between AAS and CS in 12 high-impact-factor journals in the category of ‘Clinical Medicine’.

**Methods:**

The 12 journals with the highest 2023 journal impact factor (published in June 2024), four each in General and Internal Medicine, General Surgery, and Anaesthesia, were included. Articles published in final version between January 1 and December 31, 2021 were selected, and up-to-date AAS and CS for each article were obtained on July 2, 2024 from Dimensions (https://app.dimensions.ai/discover/publication). Spearman's rank order correlations (ϱ) were used to assess the strength of the association between AAS and CS.

**Results:**

A total of 5193 outputs (2747 in Medicine, 1345 in Surgery, and 1101 in Anaesthesia) were analysed, with median (interquartile range) AAS and CS of 37 (10–157) and 16 (6–52), respectively. Medicine journals had the highest AAS and CS (124 [47–384] and 28 [8–113]), followed by Anaesthesia (12 [5–27] and 12 [5–24]) and Surgery (9 [2–24] and 11 [4–27]), respectively. There was a moderate positive correlation between AAS and CS overall (ϱ=0.589), with a moderate correlation for Medicine (ϱ=0.681) and Anaesthesia (ϱ=0.427) and a weak correlation for Surgery (ϱ=0.354) (all *P*<0.0001).

**Conclusions:**

Altmetric Attention Scores correlated with citation scores, suggesting that audience engagement via social media can influence the future impact of publications and their citation scores.

**Editor's key points**

• Alternative metrics, such as Altmetric Attention Scores, provide complementary tools for evaluating the impact of research publications in addition to established bibliometrics such as the journal impact factor.
• The journals with the highest 2023 journal impact factor, four each in General and Internal Medicine, General Surgery, and Anaesthesia, were analysed for correlations between Altmetric Attention Scores and journal impact factor.
• Altmetric Attention Scores had a moderate positive correlation with journal impact factor, and might influence the impact of publications and their citation scores.

The measure of impact following publication of a research article is determined by bibliometrics such as the citation score (CS) and the journal impact factor (JIF). The CS, or citation count, measures the number of times a published research article has been cited in the literature. These contribute towards the journal's overall JIF, which is one of the most significant metrics of scientific influence. The JIF (Clarivate™, JISC Services Ltd., Bristol, UK) is a measure of the average frequency of citations for an article in a particular journal over a 2-yr period after the year of publication. Citation-based bibliometrics have long served as a surrogate marker of the research impact of scientific publications and indicate the relative importance of a particular research output within its field.^1^, ^2^, ^3^, ^4^ However they provide limited information as they take considerable time to reflect impact in the literature.^5^

Altmetric Attention Scores (AAS) are alternative bibliometrics, developed in 2010, which consider the online impact of a published journal article.^6^^,^^7^ AAS provide real-time insight into the immediate scholarly impact of published articles based on their online attention in news outlets, scientific blogs, and public health policy documents, social media outlets such as X, Facebook, and LinkedIn, and online reference managers such as Mendeley and CiteULike.^8^^,^^9^ AAS calculations are weighted to reflect the number of mentions, the quality of the source, and the authors of the mentions.^10^ An automated algorithm produces a score that is weighted to reflect the credibility and reach of each source, with the weighting being 8 for a news article, 5 for a blog, 3 for a Wikipedia article, and 0.25 each for social media posts on outlets such as X and Facebook.^9^

The literature is conflicting regarding the association between AAS and CS. A recent systematic review of 19 articles described a range of weak-to-strong correlations between CS and AAS.^11^ However, significant heterogeneity precluded any meaningful meta-analysis.^11^ Others argue that AAS can provide an early indication of the citation success of a published article.^12^^,^^13^ Mixed results in the literature contribute to the ongoing uncertainty in how to interpret and evaluate AAS, especially among the medical community. This cross-sectional study aimed to evaluate the relationship between AAS and CS among the four highest-impact-factor (IF) journals each in General and Internal Medicine, General Surgery, and Anaesthesia.

## Methods

In this cross-sectional study, we chose to analyse publication outputs from the journals with the top four JIFs each in General and Internal Medicine, General Surgery, and Anaesthesia. IFs for 2023 (published in June 2024) were obtained from Clarivate™ Journal Citation Reports™ (https://jcr.clarivate.com/jcr/). The following journals from the category lists were excluded: *General and Internal Medicine: Nature Reviews Disease Primers* (IF 76.9) (only review articles published) and *Surgery: Endoscopy* (IF 11.5), the *American Journal of Transplantation* (IF 8.9), and the *Journal of Neurology Neurosurgery and Psychiatry* (IF 8.7) (not General Surgery journals). The journals chosen, along with their IFs, are listed in Table 1. This cross-sectional study precluded the participation of human subjects and did not meet the criteria for ‘research’ according to the HRA decision tool (https://www.hra-decisiontools.org.uk/ethics/); therefore, ethical approval was not necessary.

**Table 1:** Journals chosen, journal impact factors, total outputs, exclusions, and inclusions.

Field/journal	2023 JIF published in 2024	Total outputs in 2021 (*n*)	Outputs excluded (*n*)	Outputs analysed (*n* [% of total outputs])
General and Internal Medicine				
*Lancet*	98.4	1395	921 Abstracts 52 Case reports 27 Non-research letters and correspondence 654 Essays 2 Snapshots/images 14 News articles 126 Obituaries 4 Retractions/retraction notices 3 Errata 39	474 (34.0)
*New England Journal of Medicine*	96.2	2102	1483 Case reports 57 Non-research letters and correspondence 1218 Snapshots/images 109 Interviews 75 Videos 6 Errata 18	619 (29.4)
*British Medical Journal*	93.6	2628	1634 Non-research letters and correspondence 479 Case scenarios 47 Essays 19 News articles 1031 Patient perspectives 8 Errata 50	994 (37.8)
*Journal of the American Medical Association*	63.1	1561	901 Non-research letters and correspondence 413 Essays 51 Snapshots/images 22 News articles 306 Revisited articles 48 Errata 61	660 (42.3)
Group total	–	7686	4939	2747 (35.7)
General Surgery				
*JAMA Surgery*	15.7	401	125 Non-research letters and correspondence 108 Errata 17	276 (68.8)
*International Journal of Surgery*	12.5	280	152 Non-research letters and correspondence 141 Digests 11	128 (45.7)
*British Journal of Surgery*	8.6	544	161 Non-research letters and correspondence 131 Retractions/retraction notices 2 Snapshots/images 21 Errata 7	383 (70.4)
*Annals of Surgery*	7.5	905	347 Non-research letters and correspondence 344 Errata 3	558 (61.7)
Group Total	–	2130	785	1345 (63.1)
Anaesthesia				
*Anesthesiology*	9.1	282	90 Non-research letters and correspondence 77 Case reports 2 Obituaries 1 Errata 10	192 (68.1)
*British Journal of Anaesthesia*	9.1	488	111 Non-research letters and correspondence 108 Errata 3	377 (77.3)
*Anaesthesia*	7.5	343	95 Non-research letters and correspondence 86 Retractions/retraction notices 4 Abstract books 2 Errata 3	248 (72.3)
*Pain*	5.9	308	24 Non-research letters and correspondence 20 Errata 4	284 (92.2)
Group total	–	1421	320	1101 (77.5)
Grand total	–	11 237	6044	5193 (46.2)

### Inclusion criteria

We chose outputs from each journal that had a final publication date in 2021. This year was chosen to allow 2.5 yr years for citations to develop as it is well known that although AAS are more immediate, CS take time.^5^ Original articles, systematic reviews and meta-analyses, review articles, editorials, and research letters were included.

### Exclusion criteria

We excluded outputs that were not research letters, and those that were comments or correspondence, abstracts, abstract books, case reports, case scenarios, images or snapshots, errata, retractions or retraction notices, news articles, digests, interviews, videos, essays, revisited articles (republished historical articles), digests, patient perspectives, or obituaries.

### Search methodology

The search was performed in duplicate on July 2, 2024. The PubMed® database (https://pubmed.ncbi.nlm.nih.gov) was searched for each journal (e.g. Br J Anaesth[journal]) and limited to the years 2020–2022. This was done so that outputs from 2021 could be captured accurately. The publication list for each journal was exported to an EndNote v 20 library (Clarivate™). All outputs with a final publication date of 2020 or 2022 were excluded, and the digital object identifier (doi) numbers for outputs from 2021 were then exported to a Microsoft® Word document (Microsoft® Corporation, Redmond, WA, USA). These doi numbers were then pasted into a Dimensions search (https://app.dimensions.ai/discover/publication), and the CS and AAS were obtained on July 2, 2024 and exported to a pdf file. These scores were entered manually by one investigator into Microsoft® Excel spreadsheets (Microsoft® Corporation) and checked by another. The predetermined exclusion criteria were then applied, and outputs were selected for analysis.

### Statistical analysis

The primary outcome was the overall strength and direction of the correlation between AAS and CS, as the independent and dependant variables, respectively. Secondary outcomes were the strength and direction of the correlation between AAS and CS, by speciality field and by individual journal. The correlation between AAS and CS was initially assessed overall and then by speciality field and individual journal. A sensitivity analysis of the excluded articles was conducted in the same manner as the primary analysis. A further sensitivity analysis, of log-transformed AAS and CS, was performed using a predefined multivariable linear regression model for all included outputs, adjusted for specialty field and journal IF. Descriptive statistics were used to summarise differences between AAS and CS by speciality field and individual journal, expressed as median, interquartile range (IQR), and range. Data were assessed for normality using distribution and residual plots. For non-normal variables, Spearman's rho rank-order correlations (ϱ) were used to assess the strength of the correlations between continuous data, with the Kruskal–Wallis non-parametric test used to assess the significance of the correlations for categorical data. For the multivariable linear regression analysis, the natural logarithms of AAS (log [AAS + 1]) and CS (log [CS + 1]) were used as transformed normally distributed variables, with the Pearson's correlation coefficient (r) and r^2^ statistics used to assess correlations and the proportion of variation, respectively. The regression coefficient was expressed as beta (β) with 95% confidence intervals (CIs). Statistical significance was set at *P*<0.05, with correlation strength interpreted as negligible (ϱ/r [0.00–0.09]), weak (ϱ/r [0.10–0.39]), moderate (ϱ/r [0.40–0.69]), strong (ϱ/r [0.70–0.89]), or very strong (ϱ/r [0.90–1.00]).^14^ Data analysis was performed using STATA® SE v18.5 (StataCorp, College Station, TX, USA).

## Results

**Fig 1:**
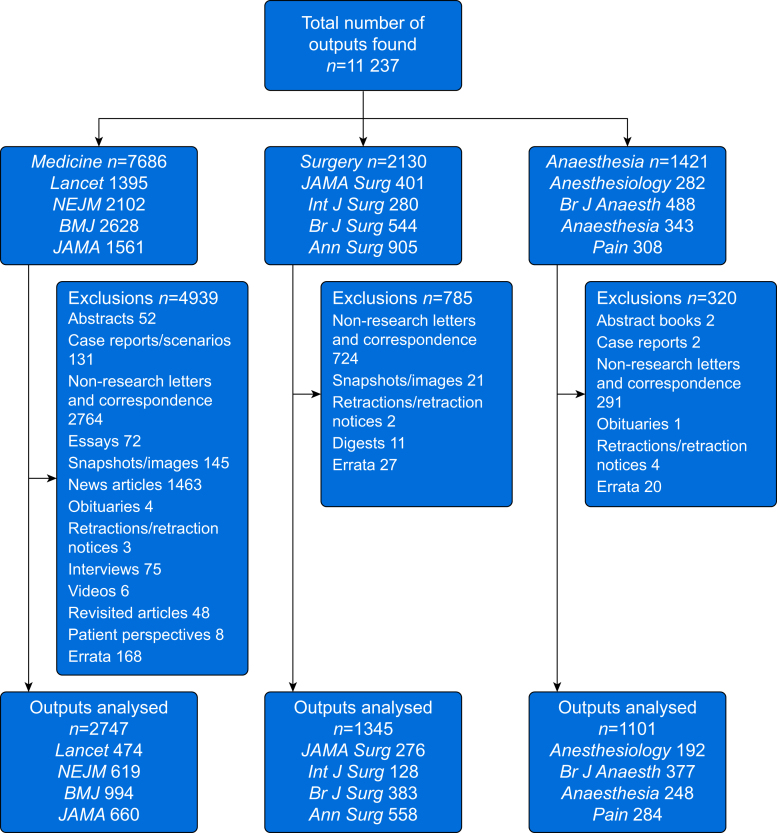
Flow diagram for inclusion and exclusion of publication outputs.

Of the 11 237 outputs identified, 5193 (46.2%) were included and available for the primary analysis, comprising 2747 (35.7%) General and Internal Medicine, 1345 (63.1%) General Surgery, and 1101 (77.5%) Anaesthesia outputs (Fig. 1 and Table 1). The highest number of included outputs (*n*=994) in 2021 was from the *BMJ*. The highest AAS and CS by speciality field were in General and Internal Medicine, with a median (IQR) of 124 (47–384) and 28 (8–113), respectively. The journal with the highest AAS and CS was the *New England Journal of Medicine*, with a median (IQR) of 249 (73–739) and 84 (16–236), respectively. The single-highest AAS and CS were 43 515 and 35 000, respectively, both for articles published in the *BMJ*.

### Primary outcome

**Fig 2:**
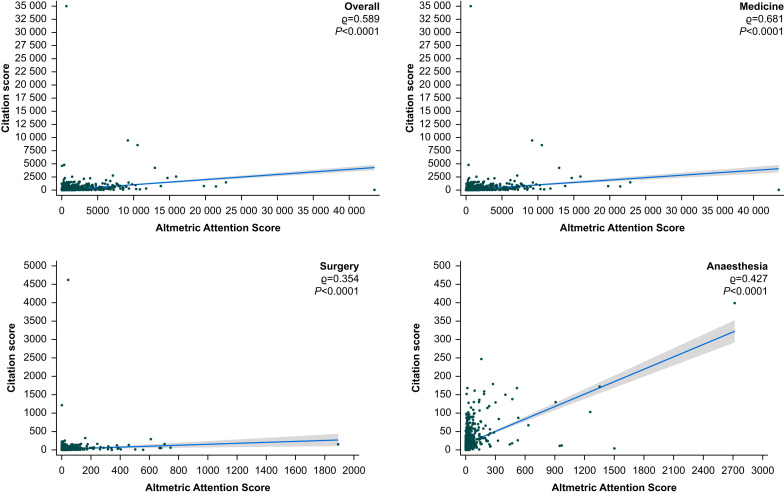
Scatter plot comparisons of Altmetric Attention Scores and Citation Scores, overall and by speciality field: overall (top left), Medicine (top right), Surgery (bottom left), and Anaesthesia (bottom right). Fitted values are for linear trend with 95% confidence intervals. *P*-values for Spearman's rank correlation coefficient (ϱ).

Of the 5193 included outputs, the overall median (IQR) AAS and CS were 37 (10–157) and 16 (6–52), respectively, with a moderate positive correlation between AAS and CS (ϱ=0.589, *P*<0.0001) (Table 2 and Fig. 2).

**Table 2:** Descriptive statistics for General and Internal Medicine, General Surgery, and Anaesthesia journals. Kruskal–Wallis tests for Medicine, Surgery, and Anaesthesia: for each field, all AAS *P*=0.0001, and all CS *P*=0.0001. AAS, Altmetric Attention Score; CS, citation score; IQR, interquartile range.

Field/journal	No. of outputs analysed	Altmetric Attention Scores			Citation Scores			Spearman's rank correlation coefficient (ϱ)	*P*-value
		Median	IQR	Range	Median	IQR	Range		
General and Internal Medicine journals									
*Lancet*	474	195	77–545	5–22 850	76.5	22–232	0–4236	0.581	<0.0001
*New England Journal of Medicine*	619	249	73–739	0–19 775	84	16–236	0–9451	0.762	<0.0001
*British Medical Journal*	994	74	30–219	0–43 515	10	3–32	0–35 000	0.593	<0.0001
*Journal of the American Medical Association*	660	114.5	50–304	1–11 742	33	11–90	0–1230	0.671	<0.0001
Medicine overall	2747	124	47–384	0–43 515	28	8–113	0–35 000	0.681	<0.0001
General Surgery journals									
*JAMA Surgery*	276	23	11–54.5	0–1892	10	2–34	0–253	0.559	<0.0001
*International Journal of Surgery*	128	0.5	0–2	0–44	8.5	3–15	0–4620	0.186	0.0356
*British Journal of Surgery*	383	10	2–22	0–610	7	3–18	0–292	0.536	<0.0001
*Annals of Surgery*	558	8	3–18	0–745	16	6–33	0–323	0.229	<0.0001
Surgery overall	1345	9	2–24	0–1892	11	4–27	0–4620	0.354	<0.0001
Anaesthesia journals									
*Anesthesiology*	192	12.5	4–32.5	0–517	9	3–27.5	0–168	0.617	<0.0001
*British Journal of Anaesthesia*	377	9	5–17	0–231	11	4–22	0–168	0.425	<0.0001
*Anaesthesia*	248	26	15.5–46.5	0–2717	11	5–25	0–399	0.519	<0.0001
*Pain*	284	7	3–21	0–949	15	8–26	0–247	0.451	<0.0001
Anaesthesia overall	1101	12	5–27	0–2717	12	5–24	0–399	0.427	<0.0001
Overall total	5193	37	10–157	0–43 515	16	6–52	0–35 000	**0.589**	<0.0001

### Secondary outcomes

#### Speciality field

Of the 2747 included General and Internal Medicine journal outputs, the median (IQR) AAS and CS were 124 (47–384) and 28 (8–113), respectively, with a moderate positive correlation between AAS and CS (ϱ=0.681, *P*<0.0001) (Table 2 and Fig. 2). Of the 1345 included General Surgery journal outputs, the median (IQR) AAS and CS were 9 (2–24) and 11 (4–27), respectively, with a weak positive correlation between AAS and CS (ϱ=0.354, *P*<0.0001) (Table 2 and Fig. 2). Of the 1101 included Anaesthesia journal outputs, the median (IQR) AAS and CS were 12 (5–27) and 12 (5–24), respectively, with a moderate positive correlation between AAS and CS (ϱ=0.427, *P*<0.0001) (Table 2 and Fig. 2). Between speciality fields, there were significant differences in AAS and CS (both *P*=0.0001) (Table 2).

**Fig 3:**
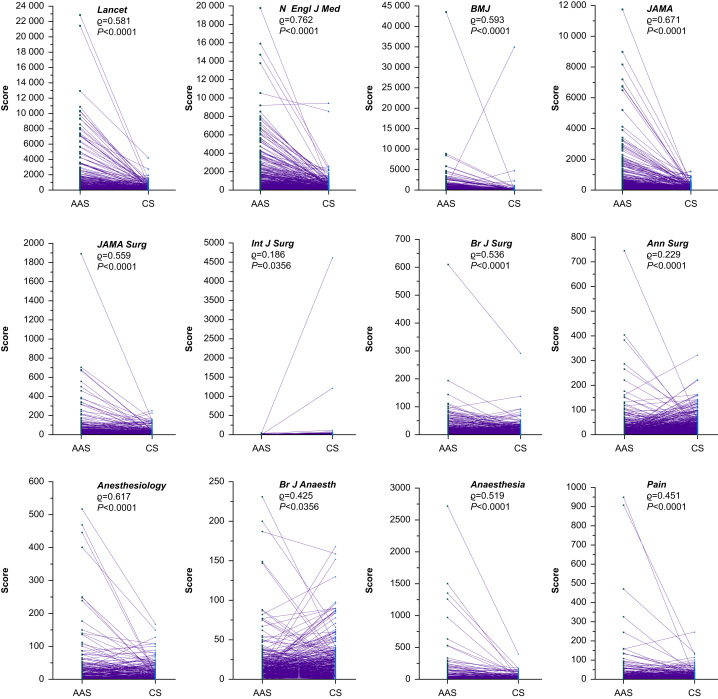
Plots of Altmetric Attention Scores and Citation Scores for individual journals. *P*-values for Spearman's rank correlation coefficient (ϱ) are shown. AAS, Altmetric Attention Score; CS, citation score.

Individual journals within the General and Internal Medicine category, all journals had a moderate-to-strong positive correlation between AAS and CS. The strongest correlation between AAS and CS was observed for the *New England Journal of Medicine* (ϱ=0.762, *P*<0.0001) and the weakest was for the *Lancet* (ϱ=0.581, *P*<0.0001) (Table 2 and Fig. 3). For the General Surgery journals, the correlation between AAS and CS ranged from weakly to moderately positive. The strongest correlation was observed for *JAMA Surgery* (ϱ=0.559, *P*<0.0001), and the weakest was for the *International Journal of Surgery* (ϱ=0.186, *P*<0.0356) (Table 2 and Fig. 3). Within the Anaesthesia category, all journals had a moderate positive correlation between AAS and CS. The strongest correlation was observed for *Anesthesiology* (ϱ=0.617, *P*<0.0001), and the weakest correlation was observed for the *British Journal of Anaesthesia* (ϱ=0.425, *P*<0.0001) (Table 2 and Fig. 3). Within each specialty field, there were significant differences between journal AAS and CS (all *P*=0.0001).

### Sensitivity analyses

#### Excluded outputs

Of the 6044 (53.8%) excluded outputs, AAS and CS ranged from 0 to 23 946 and 0 to 3593, respectively. The median (IQR) AAS and CS were 4 (1–21) and 1 (0–3), respectively, with a moderate positive correlation between AAS and CS (ϱ=0.451, *P*<0.0001). By specialty field, the correlation between AAS and CS of the excluded outputs was similar yet slightly weaker overall compared with that of the included outputs: General and Internal Medicine: *n*=4939, AAS 7 (IQR 1–28), CS 1 (IQR 0–4), ϱ=0.433, *P*<0.0001; General Surgery: *n*=785, AAS 0 (IQR 0–0), CS 0 (IQR 0–1), ϱ=0.118, *P*=0.0012; Anaesthesia: *n*=320, AAS 2 (IQR 0–4), CS 1 (IQR 0–2), ϱ=0.204, *P*=0.0003. There were significant differences between the included and excluded outputs in both AAS and CS (both *P*<0.0001).

### Linear regression

**Fig 4:**
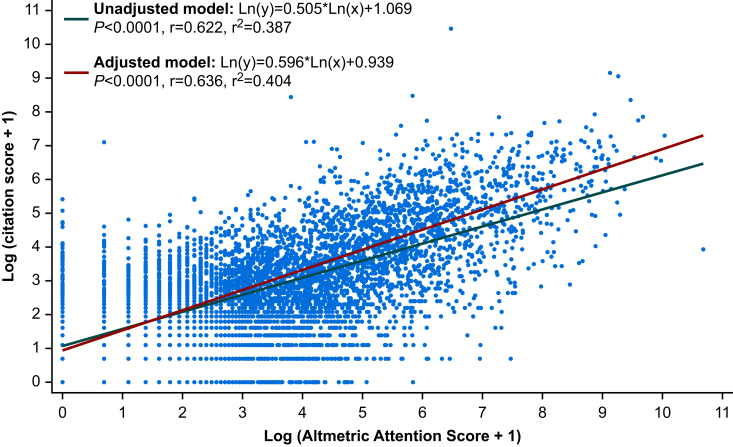
Scatter plot comparison of log-transformed overall Altmetric Attention Scores and Citation Scores. Fitted values are for the linear trend of the unadjusted (green line) and adjusted (red line) models. Correlations are expressed as Pearson's correlation coefficient (r). *P*-values for the linear regression model coefficients are shown.

Within the log-transformed overall linear regression analysis, the unadjusted model demonstrated a moderate positive correlation between AAS and CS (*n*=5193, β=0.505, 95% CI 0.488–0.523, *P*<0.0001, r=0.622) with an r^2^ of 0.387. After adjusting for specialty field and JIF, the adjusted model also demonstrated a moderate positive correlation between AAS and CS (*n*=5193, β=0.596, 95% CI 0.573–0.618, *P*<0.0001, r=0.636), with an r^2^ of 0.404, suggesting that AAS account for over 40% of the total variation observed in CS (Fig. 4).

## Discussion

This cross-sectional study demonstrated that AAS correlated with the total number of citations across 12 high-JIF journals within the fields of General and Internal Medicine, General Surgery, and Anaesthesia. The analysis included 5193 papers, of which half comprised General and Internal Medicine articles, followed by General Surgery and Anaesthesia articles. To our knowledge, this is the largest study examining the relationship between AAS and CS within Clinical Medicine and the first study to compare the speciality fields of Medicine, Surgery, and Anaesthesia. The overall median (IQR) AAS and CS were 37 (10–157) and 16 (6–52), respectively. There was a moderate positive correlation between AAS and CS for Medicine (ϱ=0.681) and Anaesthesia (ϱ=0.427), and a weak positive correlation for Surgery (ϱ=0.354) (all *P*<0.0001).

### Relationship between Altmetric Attention Scores and Citation Scores

Traditionally, the measure of impact of a research article is the total number of citations received and successful publication in a high-JIF journal. Recent evidence, however, indicates a weakening of the relationship between JIF and overall citations outside the JIF calculation period.^15^^,^^16^ Dissemination of research has been changing in recent years, with more journals offering open access publications, early availability of peer-reviewed research through published online preprints, and an increase in online-only journals. Additionally, social media outlets, such as X, have become increasingly popular outlets for disseminating research outputs to the medical community. There has been growing interest in recent years in alternative bibliometrics, such as AAS, to measure the impact of a research publication. Unlike citation counts, AAS takes into consideration the online presence and attention of articles. AAS calculations are weighted to reflect the number of mentions, the source, and the authors of the mentions.^9^ AAS provides a real-time snapshot of the impact of a publication, whereas traditional bibliometrics, such as CS, require a considerable amount of time to reflect a publication's research impact.

Previous studies have shown contradictory associations between AAS and CS.^17^, ^18^, ^19^, ^20^, ^21^ The results of this study are similar to findings from comparable studies in the current literature, and highlight the importance of the complementary role of AAS to traditional bibliometrics.^5^^,^^17^^,^^18^^,^^21^^,^^22^ However, similar analyses in the fields of plastic surgery,^19^ orthopaedics,^21^ and anaesthesia^18^ have demonstrated no significant associations. The conflicting findings are likely attributable to slight differences in the methodologies used. Importantly, this highlights the complex relationship between AAS and CS across the different fields of medicine.

### Citations and impact factors

This study has also confirmed that as JIFs are calculated as mean values, a few extremely highly cited papers can have a major influence.^23^^,^^24^ For example, while the median (IQR) CS for the *BMJ* and the *International Journal of Surgery* were 74 (30–219) and 0.5 (0–2), respectively, a single paper was cited 35 000 times in the former journal^25^ and 4620 times in the latter.^26^ Interestingly, both of these papers were the updated PRISMA guidelines for systematic reviews.^25^^,^^26^

### Strengths and limitations

Our study adds to the growing body of evidence supporting the role of alternative metrics, such as AAS, in measuring the impact of a research publication. This study has a number of strengths. Dimensions, the international database utilised for data collection, is well-recognised, large and reputable, strengthening the validity of the results presented. Only one new bibliometric, AAS, was used in the analysis, and only JIF was used to select the highest-ranking journals. Although there are other new bibliometrics, these can vary significantly between database platforms such as the Web of Science, Google Scholar, Scopus, and PubMed; for example, metrics like CiteScores and PlumX are mainly limited to journals published by Elsevier.^27^ Dimensions is the only platform that currently provides both AAS and CS for publication outputs.^28^^,^^29^ During the data collection process, two independent reviewers validated the data, increasing the reliability of the results. The large sample size gave us the power to accurately assess correlations between AAS and CS down to the individual journal level. By using the most recent 2023 JIFs with 2021 outputs, we ensured not only that our results were current but also allowed for a more appropriate assessment of AAS and CS, because of the time lag required for CS to develop compared to the immediacy of AAS, an effect previously demonstrated.^5^ Also, JIFs are calculated based on a 2-yr period, meaning the IFs used to select the highest-ranking journals would have been influenced by the data from the outputs used in this study.

By using strict inclusion and exclusion criteria, we ensured that mainly outputs included in the denominator for the calculation of JIF were assessed. Additionally, a sensitivity analysis of the excluded articles was performed to examine trends in these outputs. However, the data collected within this study are ever-changing consequent to ongoing social media usage and publications, meaning that interpretation of the results is time-specific. Although the data collected were from non-subspeciality medical disciplines, that might make the results more generalisable; the correlations described might not reflect those observed in subspeciality medical disciplines.

This study suggests that higher AAS are predictive of higher CS. However, AAS must be carefully interpreted and used as an adjunct to traditional metrics. AAS tend to be immediate, and with increasing social media activity are likely to increase in the coming years. However, unlike CS, AAS plateau once interest has waned and can even decline as a consequence of deletion of posts from social media platforms.^30^ Moreover, although AAS might provide insight into how publications influence the community and the public, ‘they lack authority and credibility as a performance measure, not least because it is easy to cheat by creating multiple accounts’, and they can also be manipulated to some extent.^31^

### Conclusions

There is a growing body of evidence supporting the use of alternative metrics, such as AAS, as a complementary tool to evaluate the impact of a research publication alongside traditional bibliometrics. This cross-sectional study suggests that AAS have a moderate positive correlation with citation scores. Despite this, AAS must be interpreted with caution. Nevertheless, audience engagement via social media might influence the future impact of publications and their citation scores.

## Authors’ contributions

Made substantial contributions to the study conception and design: AK, CAL-L, DNL

Acquisition of data: AK, TW, DNL

Analysis: CAL-L, DNL

Interpretation of data: AK, CAL-L, TW, DNL

Drafted the article and revised it critically for important intellectual content: AK, CAL-L, TW, DNL

Gave final approval of the version to be published: AK, CAL-L, TW, DNL

Agree to be accountable for all aspects of the work, thereby ensuring that questions related to the accuracy or integrity of any part of the work are appropriately investigated and resolved: AK, CAL-L, TW, DNL

## Funding

Medical Research Council (grant number MR/K00414X/1), Arthritis Research UK (grant number 19891), and the National Institute for Health Research Nottingham Biomedical Research Centre (grant number NIHR203310). The funders had no role in the design or conduct of the work, or in the decision to publish. This paper presents independent research. The views expressed are those of the authors and not necessarily those of the funders, NHS, or the Department of Health.

## Data availability statement

Data will be available upon reasonable request from the corresponding author.

## Declaration of Generative AI and AI-assisted technologies in the writing process

None used.

## Declarations of interest

None of the authors has a direct conflict of interest to report. DNL has received an unrestricted educational grant from B. Braun for unrelated work. He has also received speaker's honoraria for unrelated work from Abbott, Nestlé, and Corza.
